# Polymerase chain reaction targeting 16S ribosomal RNA for the diagnosis of bacterial meningitis after neurosurgery

**DOI:** 10.6061/clinics/2021/e2284

**Published:** 2021-01-18

**Authors:** Lauro Vieira Perdigão Neto, Micheli Medeiros, Suzete Cleusa Ferreira, Anna Shoko Nishiya, Denise Brandão de Assis, ĺcaro Boszczowski, Silvia Figueiredo Costa, Anna S. Levin

**Affiliations:** I Faculdade de Medicina (FMUSP), Universidade de Sao Paulo, Sao Paulo, SP, BR; II Laboratorio de Investigacao Medica 49, Bacteriologia, Sao Paulo SP, BR; III Departamento de Controle de Infeccao, Hospital das Clinicas (HCFMUSP), Faculdade de Medicina, Universidade de Sao Paulo, SP, BR; IV Departamento de Biologia Molecular, Fundacao Pro-Sangue / Hemocentro de Sao Paulo, Sao Paulo, SP, BR.; V Laboratorio de Investigacao Medica em Patogenese e Terapia dirigida em Onco-Imuno-Hematologia (LIM-31), Departamento de Hematologia, Hospital das Clinicas (HCFMUSP), Faculdade de Medicina, Universidade de Sao Paulo, SP, BR

**Keywords:** Meningitis, Bacterial, Craniotomy, Cerebrospinal Fluid, Diagnosis, RNA, Ribosomal, 16S, Sequence Analysis

## Abstract

**OBJECTIVES:**

Bacterial and aseptic meningitis after neurosurgery can present similar clinical signs and symptoms. The aims of this study were to develop and test a molecular method to diagnose bacterial meningitis (BM) after neurosurgery.

**METHODS:**

A 16S ribosomal RNA gene PCR-based strategy was developed using artificially inoculated cerebrospinal fluid (CSF) followed by sequencing. The method was tested using CSF samples from 43 patients who had undergone neurosurgery and were suspected to suffer from meningitis, and from 8 patients without neurosurgery or meningitis. Patients were classified into five groups, confirmed BM, probable BM, possible BM, unlikely BM, and no meningitis.

**RESULTS:**

Among the samples from the 51 patients, 21 samples (41%) were culture-negative and PCR-positive. Of these, 3 (14%) were probable BM, 4 (19%) were possible BM, 13 (62%) were unlikely BM, and 1 (5%) was meningitis negative. Enterobacterales, non-fermenters (*Pseudomonas aeruginosa* and *Acinetobacter baumannii*), *Staphylococcus haemolyticus*, *Granulicatella*, *Variovorax*, and *Enterococcus cecorum* could be identified. In the group of patients with meningitis, a good agreement (3 of 4) was observed with the results of cultures, including the identification of species.

**CONCLUSION:**

Molecular methods may complement the diagnosis, guide treatment, and identify non-cultivable microorganisms. We suggest the association of methods for suspected cases of BM after neurosurgery, especially for instances in which the culture is negative.

## INTRODUCTION

Surgeries performed on the central nervous system are complex (1). Patients undergoing such procedures often spend part of the postoperative period in intensive care unit (2).

One of the most important complications of neurosurgery is bacterial infection at the surgical site. Bacterial meningitis (BM) after neurosurgery (MAN) is a severe condition that is difficult to diagnose and manage, with mortality varying from 20% to 50% (3).

Another post-neurosurgery complication is aseptic meningitis. It mimics BM, as it also usually presents similar clinical signs and symptoms, such as neck stiffness, fever, and headache, but with negative routine bacterial cultures (4). Aseptic meningitis is caused by a postoperative inflammatory reaction through non-infectious mechanisms (5). Unlike MAN, many patients with aseptic meningitis have a self-limited course that resolves without specific therapy (6).

It is important to distinguish BM from aseptic meningitis because the uncertainty often results in unnecessary administration of antimicrobials, with consequent drug toxicity and bacterial resistance (7).

Molecular biology techniques have been widely used for the diagnosis of infectious diseases, including community-acquired BM (8). However, strategies based on polymerase chain reaction (PCR) amplification, with subsequent sequencing of the 16S ribosomal RNA (16S rRNA) for diagnosing MAN and its differential diagnoses have not been adequantely explored (9). 16S rRNA is a component of bacterial ribosomes and the most common housekeeping gene in bacteria. Sequencing has been frequently used to determine bacterial taxonomy or to evaluate the presence of bacteria in clinical samples (8,9).

The objectives of this study were to develop and test a molecular method to diagnose postoperative BM.

## MATERIAL AND METHODS

### Study design, patient inclusion, and ethics

This study evaulated an experimental assay developed at LIM-49, a research laboratory at Hospital das Clínicas, S�o Paulo, Brazil, a 2, 200-bed tertiary-care teaching hospital affiliated with the University of São Paulo, Brazil. Cerebrospinal fluid (CSF) samples, clinical data, and test results were obtained from patients admitted to the hospital.

Each patient was included only once from January 2015 to December 2016. Patient data were collected from hospital records and included age, sex, date and cause of hospitalization, underlying diseases, previous infections, current and previous use of antimicrobial drugs, date of neurosurgery (if applicable), and date of suspected meningitis (if applicable).

This study was approved by the Ethics Committee of Hospital das Clínicas (approval number 657.493). Informed consent was obtained from patients or their guardians.

### Method development

To develop a molecular method for the diagnosis of MAN, we initially inoculated sterile CSF samples with bacterial isolates. These samples were obtained from two patients without any suspicion of infection (negative culture) who required CSF removal because of normal pressure hydrocephalus.

CSF was artificially spiked with *Staphylococcus aureus* ATCC 25923 and *Escherichia coli* ATCC 25922. Serial dilutions ranged from 1.5 x 10^8^ to 1.5 x 10^1^ colony forming units (CFU)/mL.

After inoculating the samples with the strains, bacterial DNA was extracted using the MagNA Pure Compact Nuclear Acid Isolation Kit I-Large^®^ kit and the MagNA Pure Compact Instrument^®^ (Roche Diagnostics GmbH, Mannheim, Germany). The primers for regions V3 and V4 of 16S rRNA were 331F and 767R, as previously described (10,11). For PCR, buffer, MgCl_2_, HotStarTaq^®^ DNA Polymerase (QIAGEN, Hilden, Germany), dNTPs, primers, and DNase-free water were used. Amplification parameters were 95°C for 10 min, 35 cycles of DNA denaturation for 45 s at 95°C, DNA annealing for 45s at 57°C, and extension of the DNA for 2 min at 72°C. PCR products were purified using Illustra™ GFX™ PCR DNA and gel band purification kit (GE^®^, Amersham Biosciences, Piscataway, NJ) and sequenced using MegaBACE 1000 (ABI 3730 DNA Analyzer; Applied Biosystems, Alameda, CA). Sequences were analyzed using BioEdit and BLAST (http://www.ncbi.nlm.nih.gov/blast/).

### Method evaluation

After determining the feasibility of the method and the limits of detection of bacterial genetic material in the inoculated samples in the second phase of the study, CSF samples from several patients with different status concerning the possibility of meningitis were tested.

Samples from 51 patients were evaluated. These included 43 patients who had previously undergone neurosurgery (with CSF collected because of suspected meningitis or another neurological condition) and 8 patients who underwent spinal anesthesia preceding elective surgery other than neurosurgery (negative controls).

Based on the CSF results—and using classical microbiological methods—51 patients were classified into five groups (G1 to G5) according to the probability of meningitis. Patients in all groups—except G5—underwent neurosurgery. Clinical suspected meningitis was defined based on the opinion of the attending physician (considering the presence of fever, nausea, meningeal signs, and altered consciousness), CSF chemocytological data (cell count, protein, lactate, and glucose levels), and culture of CSF. G1 was confirmed BM that was evident by positive CSF culture. G2 was probable BM that was clinically suspected, with CSF count ≥500 cells/mm^3^, CSF neutrophil count ≥50%, and negative CSF culture. G3 was possible BM that was clinically suspected, with CSF count ≥500 cells/mm^3^, CSF neutrophil count <50%, and either a negative CSF culture; or clinically suspected, with CSF count <500 cells/mm^3^ and use of an antimicrobial compound for more than 1 day along with a negative CSF culture. G4 was BM unlikely, in which BM was not clinically suspected or was clinically suspected, with CSF count <500 cells/mm^3^, no use of antimicrobials (except prophylactic cefuroxime), and negative CSF culture. Finally, G5 was no meningitis, which involved no history of neurosurgery and no suspicion of meningitis (patient undergoing spinal anesthesia for elective surgery).

## RESULTS

The PCR detection limits in the inoculated samples were 1.5 x 10^2^ CFU/mL for *E. coli* and 1.5 x 10^1^ CFU/mL for *S. aureus*. Amplification occurred at all dilutions above the detection limits, and sequencing confirmed the inoculated CSF microorganisms.

Samples for 51 patients were tested. Among the 43 patients who had undergone neurosurgery, 23 (53%) were male with a mean age was 52.5 years. The most common neurological conditions that necessitated surgery were central nervous system tumors (n=12; 28%), hydrocephalus (n=6; 14%), and subarachnoid hemorrhage (n=6; 14%). All patients who underwent neurosurgery received 24 h of prophylactic cefuroxime. Among the 8 patients not treated by neurosurgery, 2 (25%) were male. The mean age was 43 years. Demographic, clinical, and laboratory data of the patients are presented in Table 1.

Of the 51 patients, 4 were G1, 5 (10%) G2, 10 (20%) G3, 24 (47%) G4, and 8 (15%) G5. In G1, the positive cultures isolated from CSF cultures were *Klebsiella pneumoniae* (n=2), *E. cloacae* (n=1), and *S. haemolyticus* (n=1). All werebut one *K. pneumoniae* were confirmed by PCR.

In the G5 control group, 7 of 8 samples were PCR-negative. Consequently, there was no molecular identification of any microorganism. In the probable (G2), possible (G3), and unlikely (G4) BM (all culture-negative) groups, 20/39 (51%) had positive PCR results. Twelve were successfully sequenced and identified. They included 3 Enterobacterales (whose species could not be specified), 3 *Variovorax* spp., 2 *Granulicatella adiacens*, 2 *P. aeruginosa*, one *A. baumannii*, and one *E. cecorum* (Table 2).

In 9 samples, two bands were present. One was at a height compatible with the band size of the 16S rRNA target gene and the other was more discreet and very close to the other band, which made the separation for sequencing impossible (Table 2).

## DISCUSSION

We developed a 16S rRNA PCR-based strategy for the diagnosis of post-neurosurgery BM. After proving the feasibility of the method and determining the detection limits, we evaluated the performance in samples from patients with different probabilities of BM. There was an excellent correlation between the positive and negative results of cultures of patients considered to have confirmed meningitis (G1) and those without meningitis (G5). In the other groups (G2, G3, and G4), microorganisms were identified in many samples, including fastidious bacteria, in many patients whose CSF culture was negative.

As previously described, we used a gram-positive coccus (*S. aureus*) and a gram-negative rod belonging to Enterobacterales (*E. coli*) because of their common use in method validation (12) and because they are representative of the etiologic agents of surgical site infections, including those of the central nervous system (13). The developed method was then applied to 51 patient samples.

In the group of patients with meningitis, there was good agreement (3 of 4) with the culture results, including the identification of species. This has been described elsewhere. In one study that enrolled patients with ventricular drainage, 91% agreement between CSF culture results and a commercial multiplex PCR assay was observed (14). It is important to point out that the one discrepant result in which a culture-positive CSF was PCR-negative could have led to not treating the patient. It is possible that PCR inhibitor substances are present, leading to false negatives, as previously described (15).

In contrast, the patient samples of the G5 negative control were almost all negative (7 of 8). The one that was not displayed an indeterminate result, which was probably a false positive, as PCR is a highly sensitive technique. Thus, any form of contamination of the sample by even trace amounts of DNA can produce misleading results (16).

In the three groups of probable, possible, and unlikely BM, some microorganisms were identified using the 16S-PCR method. Our results suggest the presence of bacteria in at least 12 of the 39 (31%) of the samples. Even in patients with unlikely meningitis, 7 of 24 (29%) samples contained well-identified microorganisms. We do not believe in the hypothesis that these are false-positive results, and that the presence of these fastidious agents indicates contamination of the samples, since in all the samples in which they were detected, they were the sole microorganisms, and all samples were accompanied by their collection, transport, and handling.

Many of the isolates detected by PCR are gram-positive and gram-negative bacteria that are easily recovered in culture, such as Enterobacterales, non-fermenters (*P. aeruginosa* and *A. baumannii*), and *S. haemolyticus*. However, *Granulicatella*, *Variovorax*, and *E. cecorum*, which were found in six samples, have rarely been implicated in human infections. This rarity of diagnosis is possibly related to the limitation of growth of the microorganisms in routine microbiology ([Bibr B17] [Bibr B18] 17-19). Many cases of post-neurosurgery meningitis remain undetectable because of the presence of non-cultivable microorganisms (7). Without molecular methods, the recognition of these agents would be unlikely.

Our study has some limitations. One is the relatively small number of cases. However, MAN ocurs relatively infrequently and few previous studies have investigated the diagnosis of this condition using molecular biology techniques. These studies are summarized in Table 3. Another limitation was the inability to identify the microorganisms in 9 samples for which the results of PCR were positive.

## CONCLUSIONS

16S-PCR and sequencing of amplicons from CSF proved to be feasible and useful in the recognition of post-neurosurgery meningitis. Traditional methods of microbiology (culture) cannot be replaced by molecular techniques, as they may identify bacteria and require an antibiogram. We believe that although the performance of the molecular method is not perfect, it may detect microorganisms that fail to grow in cultures because of low inoculum or difficulty in growth. In addition, despite limitations in the microorganism identification spectrum, methods that identify bacteria and resistance mechanisms, such as array-based methods, may be useful in the future for rapid detection of pathogens. The combination of methods brings benefits not only for positive results. If the traditional culture and the PCR-based method are both negative, a clinician may feel confident in terminating—or not initiating—antibiotic therapy. This has an impact on antimicrobial stewardship. Selective pressure for multi-resistant microorganisms can be avoided for the individual and for the hospital environment. We therefore suggest the association of methods for suspected cases of MAN, especially for cases in which the culture is negative.

## AUTHOR CONTRIBUTIONS

Perdigão Neto LV wrote the manuscript. Medeiros M, Ferreira SC, Nishiya AS, Assis DB, and Boszczowski I conceived the idea and were involved in data acquisition. Costa SF and Levin AS coordinated this study. All authors revised and accepted the final version of the manuscript.

## Figures and Tables

**Table 1:** Demographic, clinical, and laboratory data of cerebrospinal fluid samples from 51 patients examined by PCR to diagnose bacterial meningitis after neurosurgery.

Patients treated by neurosurgery (n=43)	
Male	n=23 (53%)
Age (years)	Mean: 52.5 (range:18-87)
Neurological conditions	
CNS tumors	n=12 (28%)
Hydrocephalus	n=6 (14%)
Subarachnoid hemorrhage	n=6 (14%)
Head trauma	n=5 (11%)
Cerebellar/brain hematomas	n=4 (9%)
Stroke	n=2 (5%)
Neurocysticercosis	n=2 (5%)
Cist	n=2 (5%)
Vertebral instability	n=2 (5%)
Meningioma	n=1 (2%)
Lesion in fourth ventricle	n=1 (2%)
CSF cell count	Mean: 475 cells/mm^3^ (range: 0-4904)
CSF protein level	Mean: 163 mg/dL (range: 10-735)
CSF lactate concentration	Mean: 44 mg/dL (range: 11-162)
Glucose level	Mean: 60 mg/dL (range: 3-206)

Patients treated by neurosurgery (n=43)	
Male	n=2 (25%)
Age (years)	Mean: 43 (range:39-47)

CNS: central nervous system; CSF: cerebrospinal fluid.

**Table 2:** Distribution of 51 patients in the five groups considering the probability of presenting bacterial meningitis based on the results of microbiological and molecular CSF.

Definition Number of patients	Positive by PCR	Positive by culture	PCR amplicon sequencing
G1 Confirmed bacterial meningitis n=4	3	*Klebsiella pneumoniae* (n=2) *Enterobacter cloacae* (n=1) *Staphylococcus haemolyticus* (n=1)	*K. pneumoniae* (n=1) *E. cloacae* (n=1) *S. haemolyticus* (n=1)
G2 Probable bacterial meningitis n=5	3	-	*Variovorax boronicumulans* (n=2) *Enterococcus cecorum* (n=1)
G3 Possible bacterial meningitis n=10	4	-	*Granulicatella adiacens* (n=1) *Pseudomonas aeruginosa* (n=1) *Unidentified (n=2)
G4 Bacterial meningitis unlikely n=24	13	-	*G. adiacens* (n=1) *Variovorax paradoxos* (n=1) *P. aeruginosa* (n=1) *Acinetobacter baumannii* (n=1) Enterobacterales (n=3) *Unidentified (n=6)
G5 No meningitis n=8	1	-	*Unidentified (n=1)
Total n=51	24 (47%)		

CSF: cerebrospinal fluid; *Nine samples displayed a second band in the gel, which made sequencing impossible.

**Table 3:** Main studies that used molecular methods to diagnose meningitis after neurosurgery.

Author, year	Objective	Methods	Patients (n)	Main results and conclusions
Rath, 2014	To evaluate the clinical utility of RT-PCR of CSF in combination with IL-6 and lactate and compare them to CSF culture for the diagnosis of EVD-related ventriculitis/meningitis.	Leucocyte cell count, concentrations of glucose, protein, IL-6 and lactate, RT-PCR and culture of CSF of patients with possible ventriculitis/meningitis related to EVD were performed.	42	-Agreement of 92% of culture and PCR-based method. Discrepant results were mostly considered as contaminants. -PCR-based method is suitable for the identification of microoganisms in CSF of neurosurgery patients, especially if combined with biomarkers and lactate.
Deutch, 2007	To compare a RT-PCR diagnostic strategy with culture to evaluate additional effects on diagnosis and quantification of bacterial load in ventricular drainage-related bacterial meningitis.	CSF samples from patients with EVD or ventriculoperitoneal shunt during the course of bacterial meningitis were analyzed by r16S PCR and cultures.	86	-16 patients had any positive sample in culture or PCR. Four episodes of bacterial meningitis were diagnosed via PCR alone (predominantly caused by gram-negative), five episodes via culture alone, and seven episodes via both culture and PCR. -Culture supplemented with RT-PCR may increase the number of etiologically diagnosed bacterial meningitis episodes.
Banks, 2005	To report the analysis of CSF from ventriculoperitoneal shunts or EVD using both conventional cultures and PCR.	CSF samples from patients who underwent either shunt tap or routine surveillance cultures of their ventriculostomy, with clinical suspicion of infection, were tested using standard culture techniques and r16S PCR.	28	-21% of the samples were positive using both the culture and PCR; 30% were negative in PCR and culture; 49% of the samples were culture-negative and PCR-positive. -*Cutibacterium acnes* (fomerly *Propionibacterium acnes*) and *S. aureus* were the most frequent agents. -The data suggest that PCR is a highly sensitive, rapid, and promising modality for the detection and treatment of CSF shunt ventriculostomy infection.
Druel, 1996	To evaluate the presence of bacteria in samples from patients suffering from ‘aseptic' meningitis following craniotomy.	CSF from patients suffering from post-craniotomy meningitis and negative control patients were submitted to conventional culture and to PCR using bacterial r16S universal primers, followed by phylogenetic analysis.	24	-CSF from patients with either culture-positive or culture-negative meningitis yielded positive amplifications, whereas no amplification was obtained with CSF from control patients. -Six PCR products were cloned and sequenced: *Pseudomonas* (n=3), *Escherichia* (n=2), and *Rhodococcus* (n=1). -Many cases of culture-negative (aseptic) meningitis are probably bacterial meningitis and justify antibiotic treatment.

RT-PCR: real-time polymerase chain reaction; PCR: polymerase chain reaction; IL-6: Interleukin 6; CSF: cerebrospinal fluid; EVD: external ventricular drains. r16S: 16S ribosomal RNA.
